# Chitosan-Based Sustained Expression of Sterol Regulatory Element-Binding Protein 1a Stimulates Hepatic Glucose Oxidation and Growth in *Sparus aurata*

**DOI:** 10.3390/md21110562

**Published:** 2023-10-26

**Authors:** Ania Rashidpour, Yuanbing Wu, María Pilar Almajano, Anna Fàbregas, Isidoro Metón

**Affiliations:** 1 Secció de Bioquímica i Biologia Molecular, Departament de Bioquímica i Fisiologia, Facultat de Farmàcia i Ciències de l’Alimentació, Universitat de Barcelona, Joan XXIII 27-31, 08028 Barcelona, Spain; aniyarashidpoor2017@gmail.com (A.R.); wuuanbing@gmail.com (Y.W.); 2 Departament d’Enginyeria Química, Universitat Politècnica de Catalunya, Diagonal 647, 08028 Barcelona, Spain; m.pilar.almajano@upc.edu; 3 Departament de Farmàcia i Tecnologia Farmacèutica, i Fisicoquímica, Facultat de Farmàcia i Ciències de l’Alimentació, Universitat de Barcelona, Joan XXIII 27-31, 08028 Barcelona, Spain; anna5791@gmail.com

**Keywords:** chitosan, SREBP1, gene delivery, liver, growth, *Sparus aurata*

## Abstract

The administration of a single dose of chitosan nanoparticles driving the expression of sterol regulatory element-binding protein 1a (SREBP1a) was recently associated with the enhanced conversion of carbohydrates into lipids. To address the effects of the long-lasting expression of SREBP1a on the growth and liver intermediary metabolism of carnivorous fish, chitosan-tripolyphosphate (TPP) nanoparticles complexed with a plasmid expressing the N terminal active domain of hamster SREBP1a (pSG5-SREBP1a) were injected intraperitoneally every 4 weeks (three doses in total) to gilthead sea bream (*Sparus aurata*) fed high-protein–low-carbohydrate and low-protein–high-carbohydrate diets. Following 70 days of treatment, chitosan-TPP-pSG5-SREBP1a nanoparticles led to the sustained upregulation of SREBP1a in the liver of *S. aurata*. Independently of the diet, SREBP1a overexpression significantly increased their weight gain, specific growth rate, and protein efficiency ratio but decreased their feed conversion ratio. In agreement with an improved conversion of dietary carbohydrates into lipids, SREBP1a expression increased serum triglycerides and cholesterol as well as hepatic glucose oxidation via glycolysis and the pentose phosphate pathway, while not affecting gluconeogenesis and transamination. Our findings support that the periodical administration of chitosan-TPP-DNA nanoparticles to overexpress SREBP1a in the liver enhanced the growth performance of *S. aurata* through a mechanism that enabled protein sparing by enhancing dietary carbohydrate metabolisation.

## 1. Introduction

Chitosan is a linear cationic polymer of β (1-4)-linked 2-amino-2-deoxy-d-glucose interspersed by residual 2-acetamido-2-deoxy-β-d-glucose, produced by the deacetylation of chitin under alkaline conditions [1,2,3]. The interaction between positively charged chitosan and negatively charged nucleic acids results in the spontaneous formation of polyplexes. This ability together with bioadhsesion, low immunogenicity, biodegradability, and biocompatibility, prompted recent exploration into the use of chitosan as a vector for nucleic acid delivery in fish biotechnology, as non-viral vectors enabling fish vaccination, the control of gonadal development, and the gene therapy-based modulation of fish metabolism [4]. We previously showed that a single intraperitoneal administration of chitosan-tripolyphosphate (TPP) nanoparticles complexed with plasmids to induce gene silencing or the transient expression of exogenous proteins is an efficient method to produce an acute modification of intermediary metabolism in the liver of gilthead sea bream (*Sparus aurata*). Thus, chitosan-TPP nanoparticles complexed with a plasmid encoding a short hairpin RNA (shRNA) designed to silence the expression of cytosolic alanine aminotransferase (cALT) significantly downregulated liver transamination but increased the hepatic activity of key enzymes in glycolysis [5]. The same methodology was used to demonstrate that the shRNA-mediated knockdown of glutamate dehydrogenase (*gdh*) reduced hepatic transdeamination and gluconeogenesis from amino acids but increased the metabolic use of dietary carbohydrates in the liver through the stimulation of the 6-phosphofructo-1-kinase (PFKL)/fructose-1,6-bisphosphatase (FBP1) enzyme activity ratio [6].

More recently, chitosan-TPP nanoparticles complexed with a plasmid expressing the N-terminus of hamster sterol regulatory element-binding protein 1a (SREBP1a) induced a multigenic action in the liver of *S. aurata* 72 h post-treatment, leading to the enhanced conversion of dietary carbohydrates into lipids and increased the blood levels of triglycerides and cholesterol through a mechanism involving the upregulation of the hepatic expression of genes encoding key enzymes in fatty acid and cholesterol biosynthesis, NADPH formation (glucose-6-phosphate dehydrogenase, *g6pd*), and glycolysis (glucokinase, *gck*; 6-phosphofructo-2-kinase/fructose-2,6-bisphosphatase, *pfkfb1*), while decreasing the activity of the gluconeogenic enzyme FBP1 [7].

SREBP1a belongs to the SREBP family of transcription factors. SREBPs are synthesised as inactive precursors anchored to the endoplasmic reticulum membrane. The C-terminus of SREBP precursors associates with the SREBP-cleavage activating protein (Scap), a sterol sensor, and the insulin-induced gene (Insig) protein [8,9]. Decreased cellular levels of sterol drive the SREBP/Scap complex to the Golgi apparatus, where the N-terminus of SREBPs is released after a two-step cleavage by site-1 and site-2 proteases. The N-termini of SREBPs are active basic-helix–loop–helix leucine zipper transcription factors that, after dimerisation, translocate to the nucleus and activate transcription by binding to sterol regulatory elements (SREs) in the promoter of target genes. Alternate promoters in the *srebf1* gene generate SREBP1a and SREBP1c [10,11,12,13]. The presence of a long N-terminal transactivation domain confers strong transcriptional activity to SREBP1a, which is a potent activator of all SREBP-responsive genes [14,15,16].

Given that *S. aurata* and carnivorous fish in general have low abilities to metabolise dietary carbohydrates and exhibit prolonged hyperglycaemia after a glucose load or being fed high carbohydrate diets [17,18], and the fact that a single dose of chitosan nanoparticles complexed with a plasmid encoding the N terminus of hamster SREBP1a stimulated a short-term metabolic change in the liver of *S. aurata* that enabled carbohydrate conversion into lipids [7], the aim of the present study was to study the effects of long-lasting, sustained expression of transcriptionally active SREBP1a on growth and liver intermediary metabolism in *S. aurata*. To this end, chitosan-TPP nanoparticles complexed with a plasmid expressing the N terminal active domain of hamster SREBP1a were periodically administered to *S. aurata* fed diets differing in macronutrient composition.

## 2. Results

### 2.1. Effect of Periodical Administration of Chitosan-TPP-SREBP1a on SREBP1a mRNA Levels in the Liver of S. aurata

**Figure 1:**
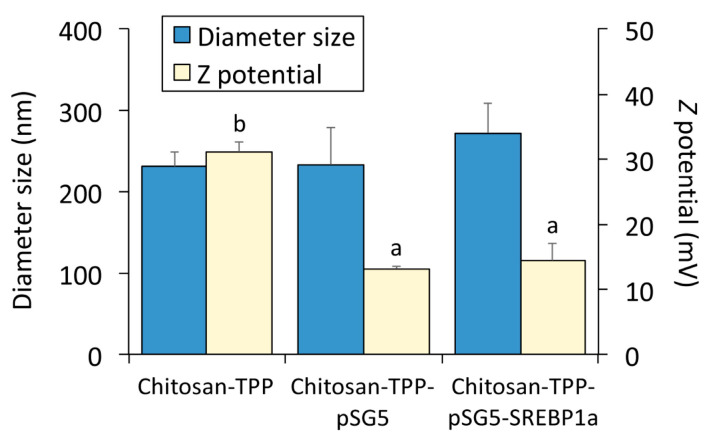
Diameter size and *Z* potential of chitosan-TPP-DNA. Particle size and *Z* potential values of chitosan-TPP nanoparticles complexed with pSG5 and pSG5-SREBP1a were determined by dynamic light scattering and laser Doppler electrophoresis. The values are expressed as mean ± SEM (*n* = 3). Different letters indicate significant differences between conditions (*p* < 0.05).

To address the effect of hepatic SREBP1a overexpression on growth and liver intermediary metabolism in *S. aurata*, chitosan-TPP nanoparticles complexed with empty pSG5 (control) and pSG5-SREBP1a were prepared by ionic gelation, as previously reported [7]. The characterisation of naked chitosan-TPP and chitosan-TPP complexed with plasmids (pSG5 and pSG5-SREBP1a) by dynamic light scattering showed no differences in particle mean diameter size. However, laser Doppler electrophoresis allowed us to show that the incorporation of pSG5 and pSG5-SREBP1a into chitosan-TPP significantly reduced the positivity of the *Z* potential to 54–58% of the values observed in naked chitosan-TPP (Figure 1).

To assess the effect of the long-term, sustained expression of SREBP1a, intraperitoneal injections of chitosan-TPP nanoparticles complexed with pSG5 or pSG5-SREBP1a were periodically administered every 4 weeks to *S. aurata*. The fish received a total of three doses of the nanoparticles (each consisting of 10 μg of the corresponding plasmid per g of body weight, BW). Sampling proceeded 14 days after the last injection, which corresponded to a total treatment period of 70 days. The dosage schedule was based on the persistent hepatic expression of exogenous enzyme activity after the chitosan-based delivery of plasmid DNA in *S. aurata* [19]. The effect of periodical administration of nanoparticles was studied in fish fed two diets differing in macronutrient composition: Diet 1 (high-protein–low-carbohydrate commercial diet) and Diet 2 (low-protein–high-carbohydrate diet).

**Figure 2:**
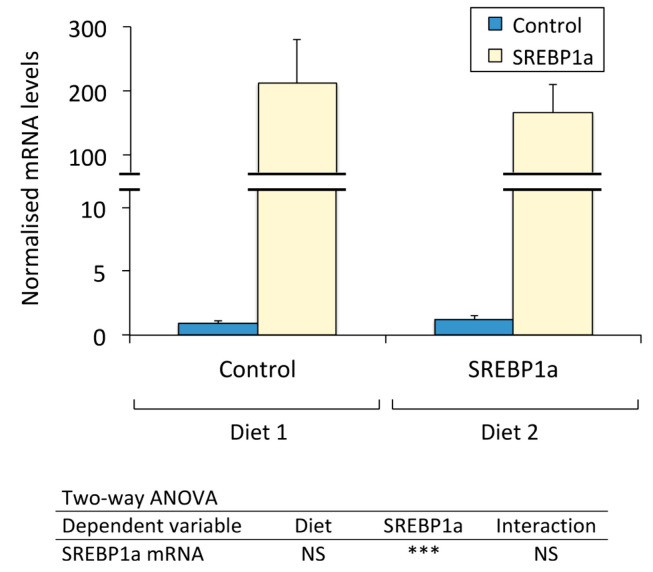
Effect of periodical administration of chitosan-TPP-DNA nanoparticles on SREBP1a mRNA levels in the liver of *S. aurata*. Three doses of chitosan-TPP nanoparticles complexed with pSG5 and pSG5-SREBP1a were periodically administered every 4 weeks by intraperitoneal injection to *S. aurata* fed Diets 1 and 2. After 70 days of treatment and 14 days following the last injection, liver samples were obtained and subjected to RNA isolation and subsequent RT-qPCR assays. The mRNA levels of SREBP1a were normalised with the geometrical mean of *S. aurata* β-actin (*actb*), elongation factor 1 alpha (*eef1a*) and ribosomal subunit 18S (*18s*). The values are expressed as mean ± SEM (*n* = 5). Statistical significance for independent variables (diet and treatment with SREBP1a nanoparticles) are indicated as follows: *** *p* < 0.001; NS, not significant.

Reverse transcription coupled with quantitative PCR (RT-qPCR) revealed a huge expression level of SREBP1a in the liver of treated fish irrespective of diet. In the liver of *S. aurata* subjected to the periodical administration of chitosan-TPP-pSG5-SREBP1a nanoparticles, the mRNA levels of SREBP1a reached 239-fold (Diet 1) and 136-fold (Diet 2) significantly higher values than in control fish (Figure 2).

### 2.2. Effect of SREBP1a Expression on Growth and Whole-Body Composition in S. aurata

Consistent with previous results, the supply of a low-protein–high-carbohydrate diet (Diet 2) significantly decreased final BW (to 86.7% of the levels found in fish fed Diet 1), weight gain (to 83.4%), and specific growth rate (SGR) (to 92.7%). In addition, higher lipid retention (LR) (2.47-fold) and protein efficiency ratio (PER) (1.14-fold) values were found in fish fed Diet 2. Independently of the diet supplied, long-term treatment with periodical administration of chitosan-TPP-pSG5-SREBP1a nanoparticles significantly increased weight gain (13.8% for fish fed Diet 1 and 9.2% for fish fed Diet 2), SGR (14.6% for fish fed Diet 1 and 3.6% for fish fed Diet 2), and PER (7.9% for fish fed Diet 1 and 20.6% for fish fed Diet 2) but decreased feed conversion ratio (FCR) (to 6.4% and 14.7% of control values in fish fed Diet 1 and Diet 2, respectively). Neither diet nor nanoparticle administration caused significant effects on hepatosomatic index (HSI), protein retention (PR), moisture, ash, protein, and lipid body composition (Table 1).

### 2.3. Effect of SREBP1a Expression on Serum Metabolites in S. aurata

**Figure 3:**
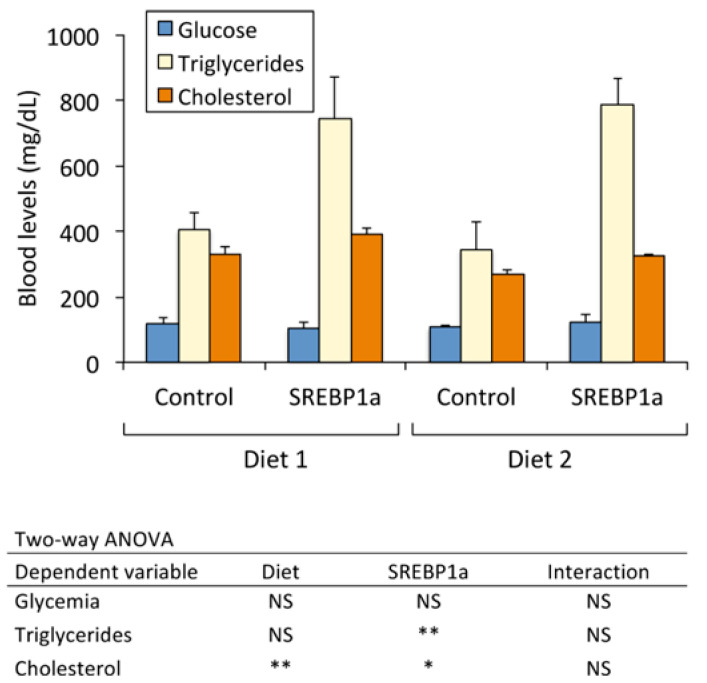
Effect of periodical administration of chitosan-TPP-DNA nanoparticles on serum glucose, triglycerides, and cholesterol in *S. aurata*. Three doses of chitosan-TPP nanoparticles complexed with pSG5 and pSG5-SREBP1a were periodically administered every 4 weeks by intraperitoneal injection to *S. aurata* fed Diets 1 and 2. After 70 days of treatment and 14 days following the last injection, blood was collected. Serum levels of glucose, triglycerides, and cholesterol are expressed as mean ± SEM (*n* = 4–7). Statistical significance for independent variables (diet and treatment with SREBP1a nanoparticles) and the interaction between independent variables are indicated as follows: * *p* < 0.05; ** *p* < 0.01; NS, not significant.

Serum glucose, triglycerides, and cholesterol were determined in 70-day treated *S. aurata* (Figure 3). Blood glucose levels remained unchanged irrespective of the treatments assayed (diet and nanoparticle administration). In contrast, the nanoparticle-mediated expression of SREBP1a in the liver of *S. aurata* significantly increased blood triglycerides and cholesterol independently of the diet supplied. Chitosan-TPP-pSG5-SREBP1a administration increased the amount of triglycerides and cholesterol by 1.8- and 1.2-fold, respectively, in fish fed Diet 1 and by 2.3- and 1.2-fold in fish fed Diet 2. In addition, total cholesterol was affected by diet, leading to 1.2-fold higher values in fish fed Diet 1.

### 2.4. Effect of SREBP1a Expression on the Intermediary Metabolism of S. aurata

**Figure 4:**
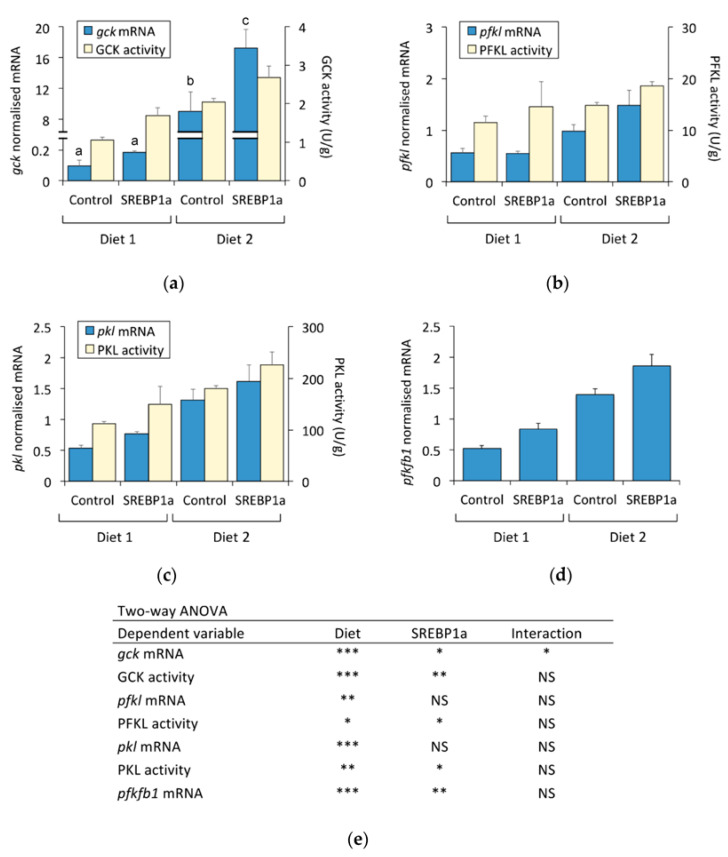
Effect of periodical administration of chitosan-TPP-DNA nanoparticles on key enzymes of liver glycolysis. Three doses of chitosan-TPP nanoparticles complexed with pSG5 and pSG5-SREBP1a were periodically administered every 4 weeks by intraperitoneal injection to *S. aurata* fed Diets 1 and 2. After 70 days of treatment and 14 days following the last injection, liver samples were obtained and subjected to RNA and crude extract isolation for subsequent RT-qPCR and enzyme activity assays. Hepatic expression of of *gck* (**a**), *pfkl* (**b**), *pkl* (**c**), and *pfkfb1* (**d**). The mRNA levels were normalised with the geometrical mean of *S. aurata actb*, *eef1a*, and *18s*. The mRNA and activity values are expressed as mean ± SEM (*n* = 5–6). (**e**) Statistical significance for independent variables (diet and treatment with SREBP1a nanoparticles) and the interaction between independent variables are indicated as follows: * *p* < 0.05; ** *p* < 0.01; *** *p* < 0.001; NS, not significant. For independent variables presenting significant interaction, different letters (a, b, c) indicate homogeneous subsets (*p* < 0.05).

The effect of the sustained expression of SREBP1a on the liver intermediary metabolism of *S. aurata* was addressed by analysing the gene expression and activity levels of key enzymes in liver glycolysis, gluconeogenesis, the pentose phosphate pathway, and amino acid metabolism. Concerning glycolysis, in addition to the gene expression of rate-limiting enzymes that control glycolytic substrate cycles, *gck*, *pfkl*, and pyruvate kinase (*pkl*) (Figure 4a–c), the activity of these enzymes was also determined given the well-known importance of their allosteric regulation. Since *pfkfb1* encodes a bifunctional enzyme that catalyses the synthesis and degradation of fru-2,6-P_2_, which in turn is a major regulator of glycolysis–gluconeogenesis [20], the mRNA levels of *pfkfb1* were also measured (Figure 4d). Dietary macronutrient composition strongly affected the gene expression and activity of glycolytic enzymes. The consumption of Diet 2 (low-protein–high-carbohydrate) significantly upregulated the expression of *gck* (94-fold), *pfkfb1* (2.6-fold), *pfkl* (1.8-fold), and *pkl* (2.5-fold), as well as the enzyme activity of GCK (2.0-fold), PFKL (1.3-fold), and PKL (1.6-fold). Treatment with chitosan-TPP-pSG5-SREBP1a significantly increased the mRNA levels of *gck* (1.9-fold for Diet 2) and *pfkfb1* (Diet 1: 1.6-fold, Diet 2: 1.3-fold) in the liver of *S. aurata*. Consistent with the rise in mRNA abundance, SREBP1a expression also produced a significant increase in GCK activity (Diet 1: 1.5-fold, Diet 2: 1.2-fold). In contrast to *gck* and *pfkfb1*, the mRNA levels of *pfkl* and *pkl* were not significantly affected by chitosan-TPP-pSG5-SREBP1a nanoparticles. However, the hepatic expression of SREBP1a significantly increased the activity of PFKL and PKL in fish fed Diet 1 and Diet 2 by 1.3-fold.

**Figure 5:**
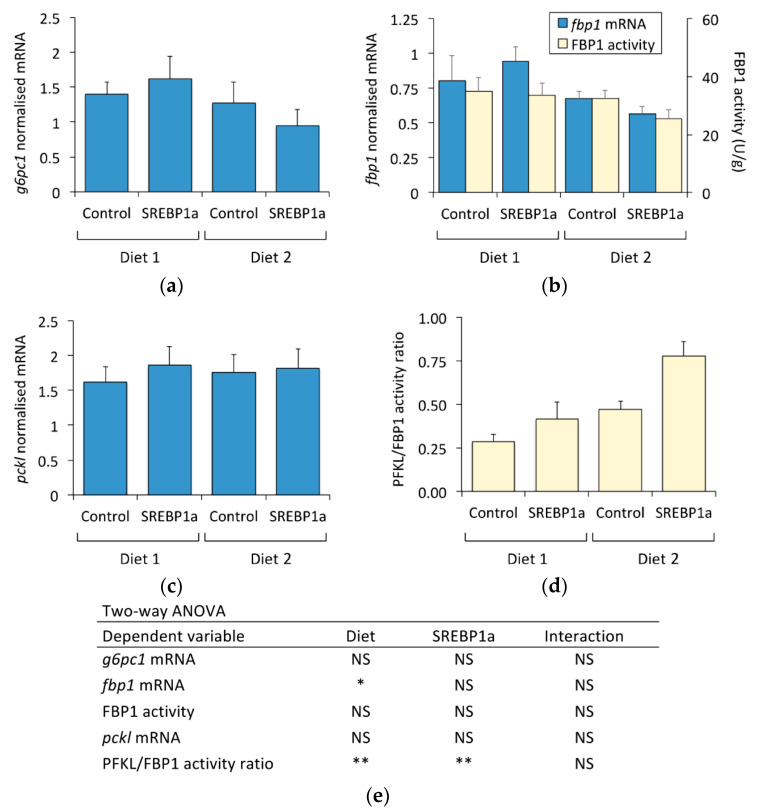
Effect of periodical administration of chitosan-TPP-DNA nanoparticles on key enzymes of liver gluconeogenesis and PFKL/FBP1 activity ratio. Three doses of chitosan-TPP nanoparticles complexed with pSG5 and pSG5-SREBP1a were periodically administered every 4 weeks by intraperitoneal injection to *S. aurata* fed Diets 1 and 2. After 70 days of treatment and 14 days following the last injection, liver samples were obtained and subjected to RNA and crude extract isolation for subsequent RT-qPCR and enzyme activity assays. Hepatic expression of *g6pc1* (**a**), *fbp1* (**b**), and *pckl* (**c**). The mRNA levels were normalised with the geometrical mean of *S. aurata actb*, *eef1a*, and *18s*. (**d**) PFKL/FBP1 enzyme activity ratio. The values are expressed as mean ± SEM (*n* = 5–6). (**e**) Statistical significance for independent variables (diet and treatment with SREBP1a nanoparticles) and the interaction between independent variables are indicated as follows: * *p* < 0.05; ** *p* < 0.01; NS, not significant.

The effect of periodical administration of chitosan-TPP-DNA on the expression of the *gck*, *pfkl*, and *pkl* counterparts in gluconeogenesis (*g6pc1*, *fbp1*, and *pckl*) was also studied. While diet composition only affected the mRNA levels of *fbp1* (1.2-fold increase in fish fed Diet 1), the chitosan-mediated expression of SREBP1a did not affect the mRNA levels of *g6pc1*, *fbp1*, and *pckl* nor FBP1 activity (Figure 5a–c). Since the fru-6-P/fru-1,6-P_2_ substrate cycle exerts a pivotal role in the control of the flux through glycolysis–gluconeogenesis, the PFKL/FBP1 activity ratio was also measured (Figure 5d). Both diet composition and SREBP1a expression significantly affected the PFKL/FBP1 activity ratio in the liver of *S. aurata*. Feeding with Diet 2 increased the PFKL/FBP1 activity ratio by 1.7-fold. Irrespective of the diet supplied, treatment with chitosan-TPP-pSG5-SREBP1a also stimulated the PFKL/FBP1 activity ratio (Diet 1: 1.5-fold; Diet 2: 1.7-fold).

**Figure 6:**
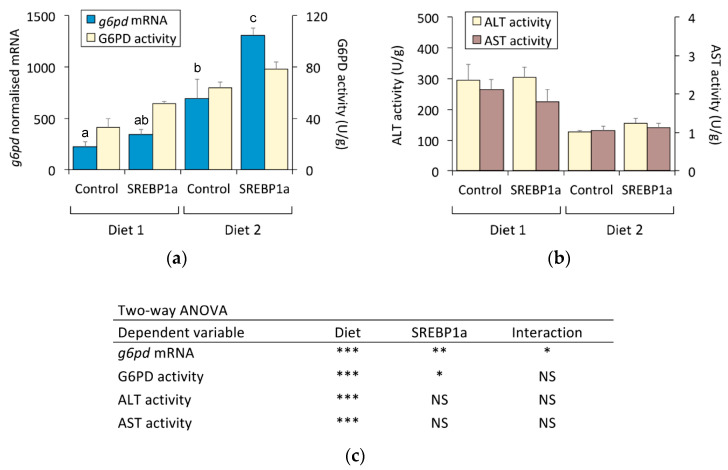
Effect of periodical administration of chitosan-TPP-DNA nanoparticles on key enzymes of liver pentose phosphate pathway and transaminases. Three doses of chitosan-TPP nanoparticles complexed with pSG5 and pSG5-SREBP1a were periodically administered every 4 weeks by intraperitoneal injection to *S. aurata* fed Diets 1 and 2. After 70 days of treatment and 14 days following the last injection, liver samples were obtained and subjected to RNA and crude extract isolation for subsequent RT-qPCR and enzyme activity assays. (**a**) Hepatic expression of *g6pd*. The mRNA levels were normalised with the geometrical mean of *S. aurata actb*, *eef1a*, and *18s*. (**b**) Liver enzyme activity of ALT and AST. The mRNA and activity values are expressed as mean ± SEM (*n* = 5–6). (**c**) Statistical significance for independent variables (diet and treatment with SREBP1a nanoparticles) and the interaction between independent variables are indicated as follows: * *p* < 0.05; ** *p* < 0.01; *** *p* < 0.001; NS, not significant. For independent variables presenting significant interaction, different letters (a, b, c) indicate homogeneous subsets (*p* < 0.05).

In addition to glycolysis and gluconeogenesis, we also explored the effect of sustained SREBP1a expression on key hepatic enzymes in the pentose phosphate pathway and transamination. Diet composition and SREBP1a expression significantly affected the mRNA levels and enzyme activity of G6PD, a rate-limiting enzyme of the oxidative phase of the pentose phosphate pathway. Feeding with Diet 2 increased *g6pd* mRNA (3.1-fold) and G6PD activity (1.9-fold), while the administration of chitosan-TPP-pSG5-SREBP1a nanoparticles significantly stimulated *g6pd* at mRNA (Diet 2: 1.9-fold) and activity (Diet 1: 1.6-fold; Diet 2: 1.2-fold) levels (Figure 6a). Diet 1 significantly increased the enzyme activity of two major liver transaminases: ALT (2.3-fold) and aspartate aminotransferase (AST) (2.0-fold). However, nanoparticle administration had no effects on the hepatic activity of ALT and AST (Figure 6b).

## 3. Discussion

The formulation of sustainable aquafeeds through the partial substitution of fishmeal by microalgae and a combination of plant- and animal-derived protein, such as soybean and poultry by-product meals, is the subject of intense research [21,22,23,24,25]. However, the substitution of fishmeal and dietary protein with cheaper nutrients with less environmental impact is a challenging issue due to the metabolic features of fish, particularly carnivorous fish, such as *S. aurata*. Carnivorous fish preferentially use amino acids as fuel and gluconeogenic substrates and therefore require high levels of dietary protein for optimal growth. Instead, carbohydrates are metabolised less efficiently than in mammals and give rise to prolonged hyperglycemia [17,18,26,27]. Nevertheless, and similarly to that previously reported [28,29], the supply of a low-protein–high-carbohydrate diet (Diet 2) induced, in the present study, metabolic adaptation in the liver of *S. aurata*, leading to the increased gene expression and activity of rate-limiting enzymes in glycolysis (GCK, PFKL, and PKL) and the oxidative phase of the pentose phosphate pathway (G6PD) but decreased the expression of the gluconeogenic gene *fbp1*.

We previously reported that the single intraperitoneal administration of chitosan-TPP nanoparticles complexed with a plasmid encoding the N-terminal active domain of hamster SREBP1a significantly upregulated SREBP1a mRNA and protein levels in the liver of *S. aurata* at 72 h post-treatment, which in turn led to increased blood triglycerides and cholesterol as a result of the enhanced hepatic upregulation of genes encoding key enzymes in fatty acid synthesis (acetyl-CoA carboxylase 1 and acetyl-CoA carboxylase 2), elongation (elongation of very long chain fatty acids protein 5), and desaturation (fatty acid desaturase 2), as well as NADPH production (*g6pd*) and cholesterol synthesis (3-hydroxy-3-methylglutaryl-coenzyme A reductase). In addition to the increased expression of genes involved in lipid biosynthesis, SREBP1a also favoured the glycolytic pathway by increasing the expressions of *gck* and *pfkfb1*, and the PFKL/FBP1 enzyme activity ratio [7]. These results led us to conclude that SREBP1a overexpression in the liver of *S. aurata* enabled dietary carbohydrate conversion into fatty acids and cholesterol.

With the aim of exploring the long-term effect of sustained expression of SREBP1a on fish growth performance and the hepatic intermediary metabolism, particularly on glycolysis–gluconeogenesis, in the present study, we periodically administered three doses of chitosan-TPP complexed with a plasmid driving the expression of the N-terminus of hamster SREBP1a (or empty plasmid as a control) to *S. aurata* fed two diets differing in macronutrient composition. After 70 days of treatment, a huge amount of SREBP1a mRNA levels were observed in the liver of fish administered with chitosan-TPP-pSG5-SREBP1a independently of the diet supplied. In agreement with previously reported persistence and stability of exogenous enzyme expression in the liver of *S. aurata* after oral delivery of chitosan-plasmid DNA polyplexes [19,30], the hepatic expression of hamster SREBP1a achieved herein after three periodical doses of nanoparticles greatly exceeded (one order of magnitude) the levels previously reported after single-dose administration [7]. Therefore, the periodical administration of chitosan-TPP-DNA nanoparticles behaved as a highly efficient nucleic acid vector to achieve long-lasting expression of an exogenous protein, following cellular uptake by endocytosis, intracellular endosomal scape of polyplexes, plasmid unpacking from chitosan, and translocation to the nucleus in fish hepatocytes.

The long-lasting expression of SREBP1a in the liver of *S. aurata* exerted a major impact on the hepatic intermediary metabolism, leading to increased circulating levels of triglycerides and cholesterol. Similarly to that for SREBP1a mRNA levels, the periodical administration of SREBP1a nanoparticles produced a greater effect on blood triglycerides, about 2-fold increase, than a single administration, which was reported to induce a 1.3-fold increase [7]. However, the sustained expression of SREBP1a did not increase the effect of a single nanoparticle dose on blood cholesterol. The rise in circulating lipids could be a direct result from the well-known transactivating effect of SREBP1 on the expression of genes involved in the biosynthesis of fatty acid and cholesterol, as it was previously demonstrated in mammals [10,13,31,32] and particularly in the liver of *S. aurata* administered with a single dose of chitosan-TPP-pSG5-SREBP1a [7].

In addition to the effect of SREBP1a on lipid metabolism, we previously showed that SREBP1a transactivates *gck* and *pfkfb1* by binding to SRE boxes in both gene promoters [33,34], while chitosan-TPP-pSG5-SREBP1a nanoparticles upregulate *gck* and *pfkfb1* mRNA levels and the PFKL/FBP1 enzyme activity ratio in the liver of *S. aurata* 72 h after nanoparticle administration [7]. The results of the present study confirm the activating effect of SREBP1a on hepatic glycolysis in *S. aurata*. The sustained expression of SREBP1a stimulated the transcriptional activation of *gck* expression, which in turn increased the intracellular content of glu-6-P and the subsequent oxidation of glu-6-P via glycolysis and the pentose phosphate pathway [35].

Further activation of the glycolytic pathway would result from SREBP1a-dependent enhanced *pfkfb1* mRNA levels. The *pfkfb1* gene encodes a bifunctional enzyme that catalyses the synthesis and degradation of fru-2,6-P_2_, which exerts a pivotal role in both glycolysis and gluconeogenesis through the allosteric activation of PFKL and the inhibition of FBP1, the enzymes that control the flux through the fru-6-P/fru-1,6-P_2_ substrate cycle [20,36,37]. Hence, the upregulation of *pfkfb1* expression and fru-2,6-P_2_ levels in the liver of *S. aurata* is associated with nutritional and physiological conditions favouring glycolysis, such as starved-to-fed transition, the supply of low-protein–high-carbohydrate diets, or treatment with glucose and insulin [28,33,38]. In the present study, the transcriptional activity of other key enzymes in glycolysis such as *pfkl* and *pkl* was not significantly affected by SREBP1a treatment. However, increased fru-2,6-P_2_ levels could be responsible for the allosteric activation of PFKL and the increase in PFKL/FBP1 activity ratio that were observed under conditions with sustained SREBP1a expression. The subsequent increase in fru-1,6-P_2_ production, which is a known allosteric activator of PKL, suggests that SREBP1a could indirectly activate PKL activity.

Similarly to that in *S. aurata*, SREBP1 isoforms have been also involved in the transcriptional activity of genes associated with glucose metabolism, favouring flux through the glycolytic pathway in the liver. For instance, SREBP1c upregulates the insulin-dependent transcription of hexokinase II and *gck* in mammals [39,40,41,42], and SREBP family members are required for the elevation of glycolysis in NK cells [43]. Furthermore, SREBP-1c silencing augmented the glucose production of HepG2 cells by upregulating the expression of *pckl* and *g6pc1* [44]. Similarly, SREBP-1 knockdown increased *pck1* expression and decreased glycogen deposition in the liver of fed mice [45], while SREBP1a transgenesis reduced gluconeogenesis in mouse liver due to the suppression of *pckl* and *g6pc1* transcription mediated by hepatocyte nuclear factor-4α [46].

The fact that the periodical administration of SREBP1a nanoparticles did not significantly affect the expression of genes encoding rate-limiting enzymes that participate in the control of key substrate cycles (*g6pc1*, *fbp1*, and *pckl*) nor did FBP1 activity support that, in contrast to glycolysis, sustained SREBP1a expression did not modulate hepatic gluconeogenesis in *S. aurata*. In regard to other important enzymes for hepatic intermediary metabolism, the present study also confirms the SREBP1a-dependent upregulation of *g6pd* expression and enzyme activity. The increased availability of glu-6-P and G6PD activity suggests an enhanced production of NADPH in the hepatocytes. Considered altogether, although *S. aurata* is a carnivorous fish with a low ability to metabolise carbohydrates, sustained SREBP1a expression in the liver seemed to trigger a long-term complex effect that favoured glucose oxidation via glycolysis and the oxidative phase of the pentose phosphate pathway to provide pyruvate, as a carbon backbone; NADPH; and ATP for hepatic lipid biosynthesis and the consequent rise in blood triglycerides and cholesterol.

Consistent with previous studies, *S. aurata* fed a low-protein–high-carbohydrate diet (Diet 2) exhibited poor growth performance [29,47]. Interestingly, in the present study, the sustained expression of SREBP1a in the liver improved the growth performance in *S. aurata* irrespective of the dietary macronutrient composition. The periodical administration of SREBP1a nanoparticles increased weight gain and SGR. Furthermore, it reduced FCR, which also indicates higher efficiency in converting feed into fish weight gain [48]. Indeed, the weight gain, SGR, and FCR values in fish treated with chitosan-TPP-pSG5-SREBP1a fed a low-protein–high-carbohydrate diet (Diet 2), were similar to those observed in the control fish (not treated with SREBP1a) fed a high-protein–low-carbohydrate diet (Diet 1). In other words, our findings support that treatment with SREBP1a compensated the loss in growth performance resulting from feeding a carnivorous fish a low-protein–high-carbohydrate diet. The fact that SREBP1a increased blood triglycerides and cholesterol while keeping the total body lipids unaffected suggests that the SREBP1a-dependent increased circulating lipids would mostly be used for growth-related structural and energy purposes rather than fat accumulation.

Treatment with SREBP1a increased PER independently of the diet supplied, which suggests that the hepatic expression of SREBP1a improved the amount of dietary protein resulting into body weight gain. Therefore, the growth-promoting effect of SREBP1a on *S. aurata* may rely on the fact that the enhanced SREBP1a-dependent metabolisation of dietary carbohydrates and subsequent conversion into lipids may favour a protein-sparing effect that enabled the use of dietary amino acids for protein biosynthesis and growth rather than as gluconeogenic substrates. Indeed, treatment with SREBP1a deeply impacted hepatic glucose metabolism without altering ALT and AST activity, which indicates that the transamination capacity of the liver remained unaffected. Consistently with the important role of SREBP1 on growth, lipogenic regulation by SREBP1 has been shown to be responsible for modulating growth in cancer cells [49,50,51,52], and SREBP proteins were reported to control the cytokine-induced growth and proliferation of natural killer cells by a mechanism involving elevated glycolysis and oxidative phosphorylation [43].

## 4. Materials and Methods

### 4.1. Experimental Design

Juvenile gilthead seabream (*S. aurata*) (7.4 g ± 0.2, mean weight ± SEM) were acquired from Piscicultura Marina Mediterranea (AVRAMAR Group, Burriana, Spain). Upon arrival to the aquatic animal facility of the Scientific and Technological Centers of the Universitat de Barcelona (CCiTUB), the fish were maintained at 20 °C in 250 L aquaria supplied with running seawater, as described [53]. For acclimation, for two weeks, the fish were fed twice daily (9:00 and 17:00) at 5% BW with Diet 1 (Dibaq Microbaq 165, Dibaq, Segovia, Spain), which is a commercial diet containing 52% protein, 18% lipids, 12% carbohydrates, 10% ash, and 21.3 kJ/g gross energy. Two weeks before nanoparticle administration and until the end of the experimental procedure, the fish were divided into 2 groups according to the experimental diet received: Diet 1 or Diet 2 (experimental low-protein–high-carbohydrate diet containing 38.6% protein, 12.1% lipids, 37.1% carbohydrates, 10.5% ash, and 20.1 kJ/g gross energy). The experimental diets were supplied at 3% BW. To study the effect of sustained SREBP1a expression on growth and intermediary metabolism, 3 intraperitoneal injections of chitosan-TPP nanoparticles complexed with pSG5-SREBP1a or empty pSG5 (each dose consisting of 10 μg plasmid per gram BW) were periodically administered every 4 weeks. Fourteen days following the last injection and 24 h after the last meal, the fish were sacrificed by cervical section; their blood was collected; and their liver was dissected out, frozen in liquid N_2_ and kept at −80 °C until use. To prevent stress, the fish were anesthetised with tricaine methanesulfonate (MS-222; 1:12,500).

### 4.2. Preparation and Characterisation of Chitosan-TPP-DNA Nanoparticles

The ionic gelation method was used to encapsulate chitosan-TPP nanoparticles with either pSG5-SREBP1a, an expression plasmid encoding the N-terminus of hamster SREBP1a [33], or pSG5 (Agilent Technologies, Palo Alto, CA, USA), which was used as a negative control (empty vector). Based on previous reports [7], 1 mg of plasmid was first mixed with 4 mL of 0.84 mg/mL TPP (Sigma-Aldrich, St. Louis, MO, USA). TPP-DNA solutions were added dropwise to 10 mL of 2 mg/mL low-molecular-weight chitosan (Sigma-Aldrich, St. Louis, MO, USA)-acetate buffer (chitosan:TPP ratio, 1:0.4). Chitosan-TPP-DNA nanoparticles were sedimented by centrifugation at 36,000× *g* for 20 min at 15 °C, rinsed twice with ultrapure water, and resuspended in 2 mL of 2% *w*/*v* mannitol (cryoprotector during lyophilisation). After a freeze–dry cycle at −47 °C, the particle size and *Z* potential of chitosan-TPP-DNA nanoparticles were determined by dynamic light scattering and laser Doppler electrophoresis, respectively, using Zetasizer Nano ZS fitted with a 633 nm laser (Malvern Instruments, Malvern, UK). Chitosan-TPP-DNA nanoparticles were resuspended in 0.9% NaCl before being administered to the *S. aurata*.

### 4.3. Growth Parameters

Weight gain is defined as the difference between final and initial body fresh weight. SGR, FCR, HSI, PR, LR, and PER were calculated according to Equations (1), (2), (3), (4), (5) and (6), respectively.

SGR = ((ln W_f_ − ln W_i_) × 100)/T, where W_f_ and W_i_ are mean final and initial body fresh weight in g and T is time in days, (1)

FCR = g dry feed intake/g wet weight gain,(2)

HSI = (g liver fresh weight × 100)/g fish body weight,(3)

PR = (g body protein gain × 100)/g protein intake,(4)

LR = (g body lipid gain × 100)/g lipid intake,(5)

PER = g weight gain/g feed protein provided,(6)

### 4.4. Whole-Body Composition

Fish were dried at 85 °C until constant weight was reached to determine moisture, which was calculated according to Equation (7) [54,55].

Moisture = (g wet weight − g dry weight) × 100/g wet weight,(7)

Dried fish were also used for assaying nitrogen (N), lipid, and ash. N content was determined using FlashEA 1112 analyser (Thermo Fisher Scientific, Waltham, MA, USA) and was multiplied by 6.25 to estimate crude protein. A Soxhlet extractor and petroleum ether were used to extract crude lipid. To determine the ash content, sample incineration was performed in a Hobersal 12PR/300 muffle furnace (Hobersal, Caldes de Montbui, Spain) at 550 °C for 12 h [54,55]. Crude protein, lipid, and ash are expressed as the percentage of dry weight.

### 4.5. Reverse Transcription Coupled to Quantitative PCR

In total, 1 μg of total RNA isolated from the powdered liver of *S. aurata* was reverse-transcribed to cDNA using random hexamer primers and Moloney murine leukaemia virus RT (Life Technologies, Carlsbad, CA, USA) for 1 h at 37 °C. A QuantStudio 3 Real-Time PCR System (Thermo Fisher Scientific, Waltham, MA, USA), was used to determine the mRNA levels of the genes listed in Table 2.

The 10 μL reaction mixtures contained 0.4 μM of each primer (Table 2), 5 μL of SYBR Green (Applied Biosystems, Foster City, CA, USA), and 0.8 μL of diluted cDNA. The temperature cycle protocol for amplification was 95 °C for 10 min, followed by 40 cycles with 95 °C for 15 s and 62 °C for 1 min. For each gene, the efficiency of the amplification reaction was determined by generating standard curves from serial dilutions of the control cDNA. Dissociation curves were run after each experiment to confirm single product amplification, and amplicon size was confirmed by agarose gel electrophoresis. To normalise gene expression, *S. aurata actb*, *eef1a*, and *18s* were used as endogenous controls. Variations in gene expression were calculated using the standard ΔΔC_T_ method [56].

### 4.6. Metabolite Determinations and Enzyme Activity Assays

Glucose, triglycerides, and cholesterol in serum were measured with commercial kits (Linear Chemicals, Montgat, Spain). The liver crude extracts to assay PFKL, PKL, FBP1, G6PD, ALT, and AST were obtained from homogenisation of powdered frozen tissue in a buffer (1:5, *w*/*v*) containing 50 mM Tris-HCl (pH 7.5), 4 mM, EDTA, 50 mM NaF, 0.5 mM phenylmethylsulfonyl fluoride, 1 mM dithiothreitol, and 250 mM sucrose. For GCK activity, the homogenisation buffer additionally contained 100 mM KCl. A PTA-7 Polytron (Kinematica GmbH, Littau-Luzern, Switzerland) was used for homogenising the samples (30 sec at 4 °C). The homogenised samples were centrifuged at 10,000× *g* for 30 min at 4 °C, and the supernatants were collected for assaying the enzyme activity. The reaction mixtures for GCK, PFKL, PK, FBP1, ALT, AST, and total protein were as previously described [57,58]. The enzyme activities were expressed as specific activity (U/g protein). One unit of GCK and PFKL activity was considered the amount of enzyme needed to consume 2 μmol of NADP^+^ or NADH, respectively, per min. One unit of PKL, FBP1, G6PD, ALT, and AST activity was defined as the amount of enzyme necessary for transforming 1 μmol of substrate per min. The metabolites and enzyme activity assays were spectrophotometrically determined at 30 °C in a Varioskan LUX multimode microplate reader (Thermo Fisher Scientific, Waltham, MA, USA).

### 4.7. Statistics

Statistical analyses were performed with SPSS Version 25 software (IBM, Armonk, NY, USA). One-way analysis of variance followed by the Scheffe post hoc test was used to analyse the nanoparticle diameter size and *Z* potential. To identify significant differences due to diet composition and nanoparticle administration with pSG5-SREBP1a or an empty vector, pSG5 (control), the experimental data were submitted to a two-way analysis of variance followed by the Scheffe post hoc test when the independent variables significantly interacted.

## 5. Conclusions

The periodical administration of chitosan-TPP-pSG5-SREBP1a nanoparticles allowed for the long-standing expression of an exogenous transcription factor in the liver of *S. aurata*. Sustained SREBP1a expression increased the hepatic glucose oxidation and circulating levels of triglycerides and cholesterol. The metabolic changes triggered by SREBP1a enhanced the metabolisation of dietary carbohydrates, leading to improved growth performance in a carnivorous fish species even when feeding low-protein–high-carbohydrate diets.

## Figures and Tables

**Table 1:** Growth performance, nutrient retention, and whole-body composition of *S. aurata* fed diets differing in macronutrient composition and after periodical intraperitoneal administration of chitosan-TPP complexed with empty vector (pSG5, control) and pSG5-SREBP1a.

	Diet 1		Diet 2		2-Way ANOVA		
	Control	SREBP1a	Control	SREBP1a	Treatment	Diet	Interaction
Initial BW (g)	9.97 ± 0.69	10.6 ± 0.8	9.41 ± 0.74	9.34 ± 0.80	NS	NS	NS
Final BW (g)	34.6 ± 1.0	38.7 ± 1.8	30.0 ± 1.5	31.8 ± 3.4	NS	*	NS
Weight gain (g)	24.7 ± 0.5	28.1 ± 0.9	20.6 ± 0.9	22.5 ± 2.7	*	***	NS
SGR (%)	1.78 ± 0.05	2.04 ± 0.04	1.65 ± 0.06	1.71 ± 0.06	*	**	NS
FCR	1.40 ± 0.03	1.31 ± 0.04	1.57 ± 0.08	1.34 ± 0.12	*	NS	NS
HSI (%)	1.55 ± 0.12	1.36 ± 0.05	1.48 ± 0.14	1.79 ± 0.10	NS	NS	NS
PR (%)	25.0 ± 3.2	19.6 ± 0.8	28.6 ± 2.7	25.5 ± 2.0	NS	NS	NS
LR (%)	24.6 ± 5.6	20.4 ± 3.4	60.8 ± 13.6	46.6 ± 4.1	NS	**	NS
PER	1.40 ± 0.03	1.51 ± 0.05	1.60 ± 0.07	1.93 ± 0.20	*	***	NS
Moisture (%)	71.3 ± 0.7	69.0 ± 2.5	71.2 ± 0.7	71.9 ± 0.3	NS	NS	NS
Ash (%)	13.7 ± 0.9	13.6 ± 0.3	13.9 ± 0.2	14.5 ± 0.5	NS	NS	NS
Protein (%)	62.2 ± 2.2	59.4 ± 2.9	61.1 ± 1.7	61.5 ± 1.1	NS	NS	NS
Lipid (%)	27.3 ± 1.4	27.6 ± 1.1	28.8 ± 1.8	27.2 ± 0.6	NS	NS	NS

Data are expressed as mean ± SEM (*n* = 3). The effect of periodical administration of chitosan-TPP complexed with pSG5 or pSG5-SREBP1a (Treatment) and diet composition (Diet) was subjected to 2-way ANOVA. Statistical significance for independent variables (treatment and diet) and the interaction between independent variables are expressed as follows: * *p* < 0.05; ** *p* < 0.01; *** *p* < 0.001; NS: not significant.

**Table 2:** Primer sequences used for RT-qPCR in the present study.

Gene	Forward Sequences (5′ to 3′)	Reverse Sequences (5′ to 3′)	GenBank Accession
*actb*	CTGGCATCACACCTTCTACAACGAG	GCGGGGGTGTTGAAGGTCTC	X89920
*eef1a*	CCCGCCTCTGTTGCCTTCG	CAGCAGTGTGGTTCCGTTAGC	AF184170
*fbp1*	CAGATGGTGAGCCGTGTGAGAAGGATG	GCCGTACAGAGCGTAACCAGCTGCC	AF427867
*gck*	TGTGTCAGCTCTCAACTCGACC	AGGATCTGCTCTACCATGTGGAT	AF169368
*g6pc1*	GCGTATTGGTGGCTGAGGTCG	AAGGAGAGGGTGGTGTGGAAG	AF151718
*g6pd*	TGATGATCCAACAGTTCCTA	GCTCGTTCCTGACACACTGA	JX073711
*pck1*	CAGCGATGGAGGAGTGTGGTGGGA	GCCCATCCCAATTCCCGCTTCTGTGCTCCGGCTGGTCAGTGT	AF427868
*pfkfb1*	TGCTGATGGTGGGACTGCCG	CTCGGCGTTGTCGGCTCTGAAG	U84724
*pfk1*	TGCTGGGGACAAAACGAACTCTTCC	AAACCCTCCGACTACAAGCAGAGCT	KF857580
*pklr*	CAAAGTGGAAAGCCGGCAAGGG	GTCGCCCCTGGCAACCATAAC	KF857579
*srebp1a*	CCTCCTGCCTCCGAGTTTCC	GAAGGAAGGCTAGAATACCCC	U09103
*18s*	TTACGCCCATGTTGTCCTGAG	AGGATTCTGCATGATGGTCACC	AM490061

## Data Availability

The data presented in this study are available from the corresponding author upon request.
